# Accuracy of a new rapid diagnostic test for urinary antigen detection and assessment of drug treatment in opisthorchiasis

**DOI:** 10.1186/s40249-023-01162-4

**Published:** 2023-11-21

**Authors:** Chanika Worasith, Jiraporn Sithithaworn, Phattharaphon Wongphutorn, Chutima Homwong, Kanoknan Khongsukwiwat, Anchalee Techasen, Kulthida Y. Kopolrat, Watcharin Loilome, Nisana Namwat, Bandit Thinkamrop, Chaiwat Tawarungruang, Attapol Titapun, Thewarach Laha, Ross H. Andrews, Simon D. Taylor-Robinson, Paiboon Sithithaworn

**Affiliations:** 1 Department of Adult Nursing, Faculty of Nursing, Khon Kaen University, Khon Kaen, Thailand; 2 Cholangiocarcinoma Research Institute, Khon Kaen University, Khon Kaen, Thailand; 3 Faculty of Associated Medical Sciences, Khon Kaen University, Khon Kaen, Thailand; 4 Biomedical Science Program, Graduate School, Khon Kaen University, Khon Kaen, Thailand; 5 Faculty of Public Health, Kasetsart University Chalermphrakiat Sakon Nakhon Province Campus, Sakon Nakhon, Thailand; 6 Department of Systems Biosciences and Computational Medicine, Faculty of Medicine, Khon Kaen University, Khon Kaen, Thailand; 7 Khon Kaen University Phenome Centre, Khon Kaen University, Khon Kaen, Thailand; 8 Department of Epidemiology and Biostatistics, Faculty of Public Health, Khon Kaen University, Khon Kaen, Thailand; 9 Department of Surgery, Faculty of Medicine, Khon Kaen University, Khon Kaen, Thailand; 10 Department of Parasitology, Faculty of Medicine, Khon Kaen University, Khon Kaen, Thailand; 11 Faculty of Medicine, St Mary’s Campus, Imperial College London, London, UK; 12 Division of Digestive Health, Department of Surgery and Cancer, Imperial College London, London, UK

**Keywords:** Liver fluke, *Opisthorchis viverrini*, Urinary antigen detection, Urinary *Opisthorchis viverrini* rapid diagnosis test, Enzyme-linked immunosorbent assay, Quantitative formalin-ethyl acetate concentration technique

## Abstract

**Background:**

Screening for opisthorchiasis, a parasitic worm infection affecting many millions of people in Southeast Asia, has traditionally relied on faecal egg examination such as the formalin-ethyl acetate concentration technique (FECT) and Kato-Katz method. Although the urinary enzyme-linked immunosorbent assay (ELISA) has been used more recently, we developed a urinary antigen-based rapid diagnostic test (RDT) to simplify diagnosis and as a point-of-care testing (POCT) and field applications for surveillance and control of opisthorchiasis.

**Methods:**

A urinary *Opisthorchis viverrini* (OV)-RDT was developed using immunochromatographic methodology with a specific monoclonal antibody against OV. The diagnostic performance of the urinary OV-RDT was compared to that of quantitative faecal FECT and urinary antigen ELISA (*n* = 493). Cross-reactivities of urinary OV-RDT with other helminthiases coexisted with *O. viverrini* were determined (*n* = 96). A field trial in the application of urinary OV-RDT was compared with urinary antigen ELISA at baseline screening and assessment of drug treatment outcomes in opisthorchiasis (*n* = 1629). The McNemar chi-square, Kruskal–Wallis and Cohen’s kappa coefficient (*κ*-value) tests were used for statistical analyses.

**Results:**

Urinary OV-RDT had sensitivity of 94.2% and specificity of 93.2%, compared to faecal FECT. Urinary OV-RDT had high diagnostic agreement (Kappa = 0.842–0.874, *P* < 0.001) and quantitative correlation with urinary antigen ELISA (Kruskal–Wallis tests = 316.2, *P* < 0.0001) and faecal FECT (Kruskal–Wallis tests = 362.3, *P* < 0.0001). The positive rates by OV-RDT, ELISA and FECT were 48.9%, 52.5% and 49.3%, respectively. Cross-reactions of urinary OV-RDT with other helminthiases were few (2%). Field trials of urinary OV-RDT yielded comparable prevalence of *O. viverrini* between urinary OV-RDT (53.2%) and urinary antigen ELISA (54.0%). OV screening showed high diagnostic agreement (kappa > 0.8, *P* < 0.0001) between urinary OV-RDT and urinary antigen ELISA. The cure rates of opisthorchiasis at 1 month post-praziquantel treatment determined by urinary OV-RDT (86.6%) and urinary antigen ELISA (80.5%) were similar (*P* > 0.05).

**Conclusions:**

The urinary OV-RDT test has high potential as a new tool for screening and evaluating treatment outcomes in opisthorchiasis. The ease of sample collection and simplicity of urinary OV-RDT may facilitate mass screening, control and elimination of opisthorchiasis, thereby contributing to a reduction in the disease burden in Southeast Asia.

**Graphical Abstract:**

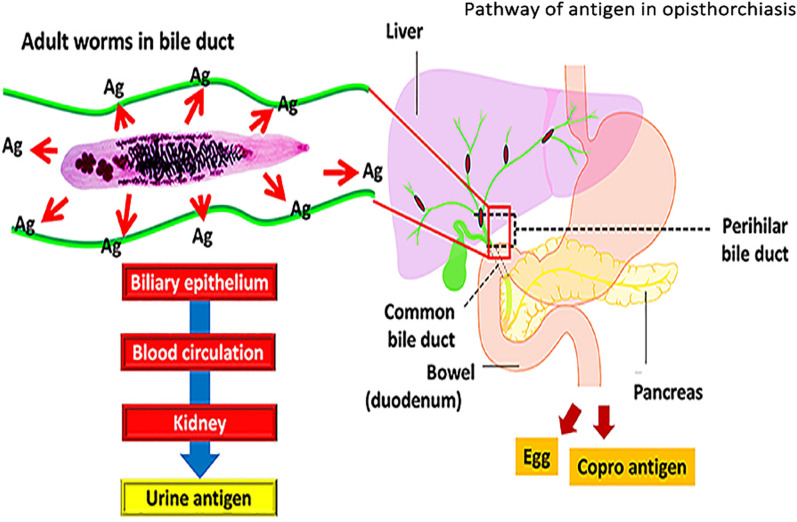

**Supplementary Information:**

The online version contains supplementary material available at 10.1186/s40249-023-01162-4.

## Background

Opisthorchiasis due to the helminth, *Opisthorchis viverrini*, is recognized by the World Health Organization (WHO) as a neglected tropical disease, which is prevalent in countries of Southeast Asia with high disease burden, particularly Thailand [1–3]. To complete the life cycle*, O. viverrini* needs three hosts, including freshwater snails (*Bithynia* spp.) to produce cercariae, fresh water fish (cyprinid) to produce metacercariae, and mammalian hosts, including humans and domestic animals, to develop adult flukes in the bile ducts [4]. The deeply embed practice of consumption of raw or undercooked freshwater fish is the principal mode of infection [5–7]. *O. viverrini* is classified as a group 1 carcinogen, as it is associated with cholangiocarcinoma (CCA) development, resulting in high mortality rates [8–10]. Among control options, primary prevention is preferable to reduce the risk of CCA from liver fluke infection [10, 11]. This approach requires a sensitive and practical method of diagnosis for *O. viverrini* surveillance and control of opisthorchiasis.

Currently, standard diagnosis of *O. viverrini* infection depends on conventional parasitological methods to detect eggs in faeces, but these are unreliable and require experienced microscopists who are in short supply [12]. A long-standing mass drug administration has resulted in most people still having light *O. viverrini* infection, but faecal examination methods have low diagnostic sensitivity [13]. Moreover, faecal egg examination is not able to detect prepatent infection, obstruction of eggs in the biliary tract due to chronic fibrosis, or blockage by adult worms following recent anthelmintic treatment [12–14]. Repeated stool examination is recommended to increase the reliability of faecal examination [15, 16], but this further increases logistical complexity during population screening. To date, several methods, including molecular and immunological approaches, have been developed to help overcome the difficulty of differentiating *O. viverrini* eggs from those of other fish-borne trematodes [12, 13, 17]. Antigen detection approaches using a monoclonal antibody-based enzyme-linked immunosorbent assay (ELISA) have been developed in opisthorchiasis with copro-antigens [18, 19], and recently, there has been improved sensitivity [20].

Subsequently, a novel diagnostic urinary antigen detection method was established for opisthorchiasis that allows more convenient sample collection and provides higher diagnostic accuracy than faecal examination [21, 22]. Urine assays yielded qualitative and quantitative diagnostic concordance with faecal egg counts [21, 22]. Without praziquantel anthelminthic treatment, the *O. viverrini* urinary antigen persisted for several months, which diminished after curative therapy [23, 24].

However, the urinary antigen assay relied on the ELISA method which requires processing time and well-equipped laboratories, hence, it is not suitable for usage in the field or resource-poor settings. In order to facilitate the use of antigen detection in opisthorchiasis, it is necessary to simplify and shorten the test time for the urine antigen assay.

The objective of this study was to develop a rapid diagnostic test for the detection of *O. viverrini* antigen in urine (OV-RDT) using monoclonal-based immunochromatographic techniques (ICTs). The performance of urinary OV-RDT was evaluated compared to standard faecal egg examination by the quantitative formalin-ethyl acetate concentration technique (FECT) and urinary antigen ELISA. We therefore aimed to evaluate the performance of urinary OV-RDT in a field screening trial program and in a treatment follow-up study to measure cure rates of praziquantel treatment. The specificity of urinary OV-RDT was evaluated by determining cross-reactivity with other parasitic infections.

## Methods

### Sample populations and study areas

The sample populations in this study were divided into three groups. Group 1 was assigned for evaluation of urinary OV-RDT performance: well-defined faecal-positive and -negative individuals for *O. viverrini* (*n* = 493) from endemic areas in northeast Thailand (samples were obtained from the Department of Parasitology Biobank, Khon Kaen University, Thailand). The sample population in group 2 was selectively recruited for cross-reactivity assessment of urinary OV-RDT with other helminthiases identified by faecal examination (*n* = 96). The sample populations in group 3 were individuals participating in an ongoing prospective field trial and a treatment follow-up study on opisthorchiasis. These participants were residents from subdistricts of Khon Kaen, Maha Sarakham and Roi Et provinces of northeast Thailand with a known high prevalence of *O. viverrini* infection (*n* = 1629) (Table 1). The study was conducted from March 2020 to December 2022.

**Table 1:** Sample populations employed for diagnostic accuracy and cross-reactivity studies (group 1–2) and field testing of urinary OV-RDT (group 3)

Study population Group	Sample number	OV+	OV−	Sampling/diagnostic method	Purpose
1	493	243	250	FECT	Accuracy of OV-RDT
2	96	0	96	Helminth infections^a^	Cross-reactivity studies
3	1629	866	763	Baseline and follow up post treatment	Field trial and evaluation of drug treatment

OV, *Opisthorchis viverrini*; OV-RDT, *Opisthorchis viverrine*-rapid diagnosis test; FECT, Formalin-ethyl acetate concentration technique

^a^Other helminths included *Strongyloides stercoralis,* minute intestinal flukes (MIFs), hookworms, *Taenia* spp., *Echinostoma* sp*.* and* Trichuris trichiura*

### Clinical sample collection and processing

All participants provided demographic data and clinical specimens. Both faecal and urine samples were collected on the same day. For faecal samples, 2 g of fresh stool was preserved in 10% formalin and processed by FECT as a reference test. Urine samples were kept on ice for transportation to the laboratory at Khon Kaen University and were centrifuged at 500×*g* for 15 min at 4 °C to separate supernatants and stored at − 20 °C for analysis by urinary OV-RDT and ELISA.

### Formalin-ethyl acetate concentration technique (FECT)

The quantitative FECT was used for faecal examination in this study because it has advantages over other methods in using fresh or formalin-preserved faecal sample, it facilitates the discrimination of *O. viverrini* and minute intestinal flukes (MIF; *Phaneropsolus*/*Prosthodendrium*) and it is sensitive for light infections as well as for *Strongyloides* infections [12, 25]. For quantitative diagnosis, FECT yielded faecal egg counts comparable to Kato-Katz method and correlated with worm burden, and also antigen concentration in urine [22, 24–26]. The procedures for quantitative FECT were used as described previously [21]. Briefly, 2 g of fresh or preserved stool samples were homogenized, filtered, and centrifuged, and ethyl acetate was added for extraction of fat from the stool. The faecal suspension was centrifuged at 500 × *g* for 5 min, and the supernatant was discarded and the resulted sediment fixed with 1 ml of 10% formalin. The final faecal suspension was examined with three drops of 40 µl per sample using a compound microscope. The mean number of eggs was multiplied by the number of drops in the suspension, and divided by the mass of stool in grams to calculate the number of eggs per gram of faeces (EPG) [14].

### Antigen preparation

In this study, the crude somatic antigen from adult *O. viverrini* was used. The antigen extraction procedure previously published was followed [27]. In brief, grossly undamaged worms obtained from the biliary system of infected hamsters were washed with sterile phosphate buffer saline (PBS) pH 7.2 and homogenized in glass tissue grinder at 4 °C in a small volume of PBS in the presence of 1 × Protease inhibitor Cocktail (Calbiochem, CA, USA). The worm homogenates were ultrasonicated and kept overnight at 4 °C before being centrifuged at 5000×*g* for 30 min. The supernatant was used as somatic antigen and kept in small aliquots at − 20 °C. The protein contents were measured by the Bradford’s method [28].

### Mouse immunization

Five-week-old male BALB/c mice were immunized a total of 3 times with 25 μg (200 μl) of crude *O. viverrini* at a concentration of 0.5 mg/ml in an equal volume of Freund’s complete (first immunization) or incomplete (second and third immunizations) adjuvants administered subcutaneously at three-week intervals. The final immunization was undertaken with 25 μg of crude *O. viverrini* in normal saline solution (NSS). Serum samples were collected prior to the immunizations for the analysis of antibody titers. Mice were housed in the specified pathogen-free animal facility at Northeast Laboratory Animal Center, Khon Kaen University.

### Hybridoma production

Three days after the last immunization, mice were sacrificed to remove the spleens for single cell preparation. A single cell suspension was prepared by pressing the spleen with syringe plug that was placed over a metal mesh in serum-free RPMI 1640 medium on a sterile petri dish, and carefully disaggregated using a cell strainer to form a single-cell suspension. The spleen cell suspension was washed three times with cold serum-free RPMI 1640 medium and centrifuged at 400 × *g* at 4 °C for 5 min followed by 3 washes using a serum-free medium (SFM) (Thermo Fisher Scientific, Waltham, MA, USA). The cells were mixed at a ratio of 1:5 viable parental myeloma cells to each viable splenocyte, and carefully fused using 1 ml of polyethylene glycol (PEG) (Sigma-Aldrich, St. Louis, MO, USA) for 1 min. The fused cells were resuspended in SFM and incubated in a water bath at 37 °C for 15 min, serum containing medium (SCM; 10% of fetal bovine serum in SFM), placed in a T-75 cm^2^ tissue culture flask (Sigma-Aldrich, St. Louis, MO, USA) containing 20 ml of 10% SCM (total culture volume is 30 ml), and incubated at 37 °C and 5% CO_2_ overnight. The fused cell suspension was removed from the flask, centrifuged, and transferred to a bottle containing 90 ml of ClonaCell™-HY Medium D (STEMCELL Technologies, Waterbeach, Cambridge, UK) and mixed thoroughly. The Medium D-containing fused cells were carefully transferred to the Petri dishes (1 × 10 cm). The dishes containing fused cells were incubated at 37 °C and 5% CO_2_. After 14 days, colonies detected on each Petri dish were transferred into an individual well of a 96-well tissue culture plate (Sigma-Aldrich, St. Louis, MO, USA) containing 200 μl of selection media (5% of fetal bovine serum in SFM with 1 × hypoxanthine, aminopterin, and thymidine (HAT; Sigma-Aldrich, St. Louis, MO, USA), and the plates were incubated at 37 °C and 5% CO_2_ for 3–4 days prior to preliminary screening by indirect ELISA.

### Screening of hybridoma cells by indirect ELISA

Screening of monoclonal antibody clones was performed by indirect ELISA. The flat-bottomed microtiter plates (Maxisorp, NUNC, Denmark) were coated overnight at 4 °C with 100 μl/well of 5 μg/ml crude *O. viverrini* in carbonate buffer pH 9.6. The plates were washed three times with normal saline containing 0.05% Tween 20 (NSST) and were blocked with 200 μl/well 5% skim milk in carbonate buffer pH 9.6 at 37 °C for 1 h. The plates were then washed as above, and 100 μl/well of hybridoma culture medium was added after which the plate was incubated at 37 °C for 1 h. Following washing, bound antibodies were detected by the addition of 100 μl/well of HRP-conjugated rabbit anti mouse IgG (Invitrogen, CA, USA), diluted at 1:2000 in 2% skim milk in PBS containing 0.05% Tween 20 at 37 °C for 1 h. After washing, 100 μl/well of 0.04% OPD in citrate phosphate buffer pH 5.0 was added and incubated at RT for 20 min. The reaction was stopped with 100 μl/well of 4 mol/L H_2_SO_4_ and the plates were read at 492 nm. Pre-immunization mouse sera and the sera from mice that received the full course of immunization (100 μl, diluted 1:1000 in PBST) were used as negative and positive controls, respectively.

### Production and purification of monoclonal antibodies

The antibody isotype of the final hybridoma cell line (OV-3) was determined by a Pierce Rapid ELISA Mouse mAb isotyping Kit (Invitrogen, CA, USA). The isotyping results showed that the clone was the mouse immunoglobulin subclass 2b (IgG2b) with kappa as the light chain. The monoclonal antibody was produced by in vitro culture of the hybridoma cell line. It was adapted to hybridoma serum-free media (HSFM) (Invitrogen, CA, USA) and cultured in a 1000 ml WHEATON® CELLine™ flask (Taylor Scientific, St. Louis, MO 63144, USA) using HSFM. The highly concentrated monoclonal antibody was harvested, and fresh media was added for media replacement. The viability of hybridoma cells was determined by trypan blue dye exclusion assay. Culture supernatants were pooled and were purified using a HiTrap® IgG Purification HP (GE Healthcare Bio-Sciences, Uppsala, Sweden) attached to an AKTATM start chromatography system (GE Healthcare Bio-Sciences, Uppsala, Sweden) following the manufacturer’s instructions. Briefly, the cell supernatant was precipitated with saturated (NH_4_)_2_SO_4_ to a final concentration of 0.8 mol/L. The ammonium sulfate-containing supernatant was filtered through a 0.45 µm filter immediately before applying it to the column. Before applying the sample to the column, the column was washed with 5 ml of distilled water to remove ethanol, then the column was equilibrated with 5 ml of binding buffer [20 mmol/L sodium phosphate and 0.8 mol/L (NH_4_)_2_SO_4_ with pH 7.5] at a flow rate of 1 ml/min. The sample was applied to the column at a flow rate of 1 ml/min. Eluted and collected the bound proteins (IgG) with 10 ml elution buffer. The unbound sample was washed out using 15 ml of binding buffer with a flow rate of 1 ml/min until the absorbance reached a steady baseline. After elution, the column was regenerated and washed with 7 ml of wash buffer (20 mmol/L sodium phosphate, pH 7.5 with 30% isopropanol) and re-equilibrated with 5 ml of binding buffer, prior to the subsequent purification. Finally, the IgG isolated was kept at -80 ℃ for urine ELISA and urinary OV-RDT.

### Monoclonal antibody-based urinary antigen ELISA

The protocol for *O. viverrini* urinary antigen measurement by ELISA was slightly modified from previous descriptions [21, 22]. The main modifications were the use of monoclonal antibody cell line (OV-3) and shortened incubation times to 45 min for urine samples, captured rabbit IgG antibodies and biotinylated anti-rabbit IgG conjugates, while the rest of the procedures remained the same.

Known cases of *O. viverrini* infection-negative and -positive urine samples (*n* = 20) determined by faecal FECT were used to construct a receiver operation curve (ROC). The cut-off points (OD value was 0.31) for diagnosis by urine ELISA was calculated using MedCalc software version 9.6.3 (MedCalc, Ostend, Belgium). The standard curves for the calculation of antigen concentrations in urine samples were constructed based on spiked urine with crude antigen of *O*. *viverrini* by ELISA. The relationships between the urinary concentrations (X) and OD values (Y) from ELISA were estimated by the best-fit linear regression equation of Log Y = 0.767X − 0.854. From the cut-off of OD = 0.31, the calculated cut-off value of OV antigen in urine became 32.9 ng/ml.

### Opisthorchis viverrini rapid diagnostic test for opisthorchiasis (urinary OV-RDT)

**Fig. 1:**
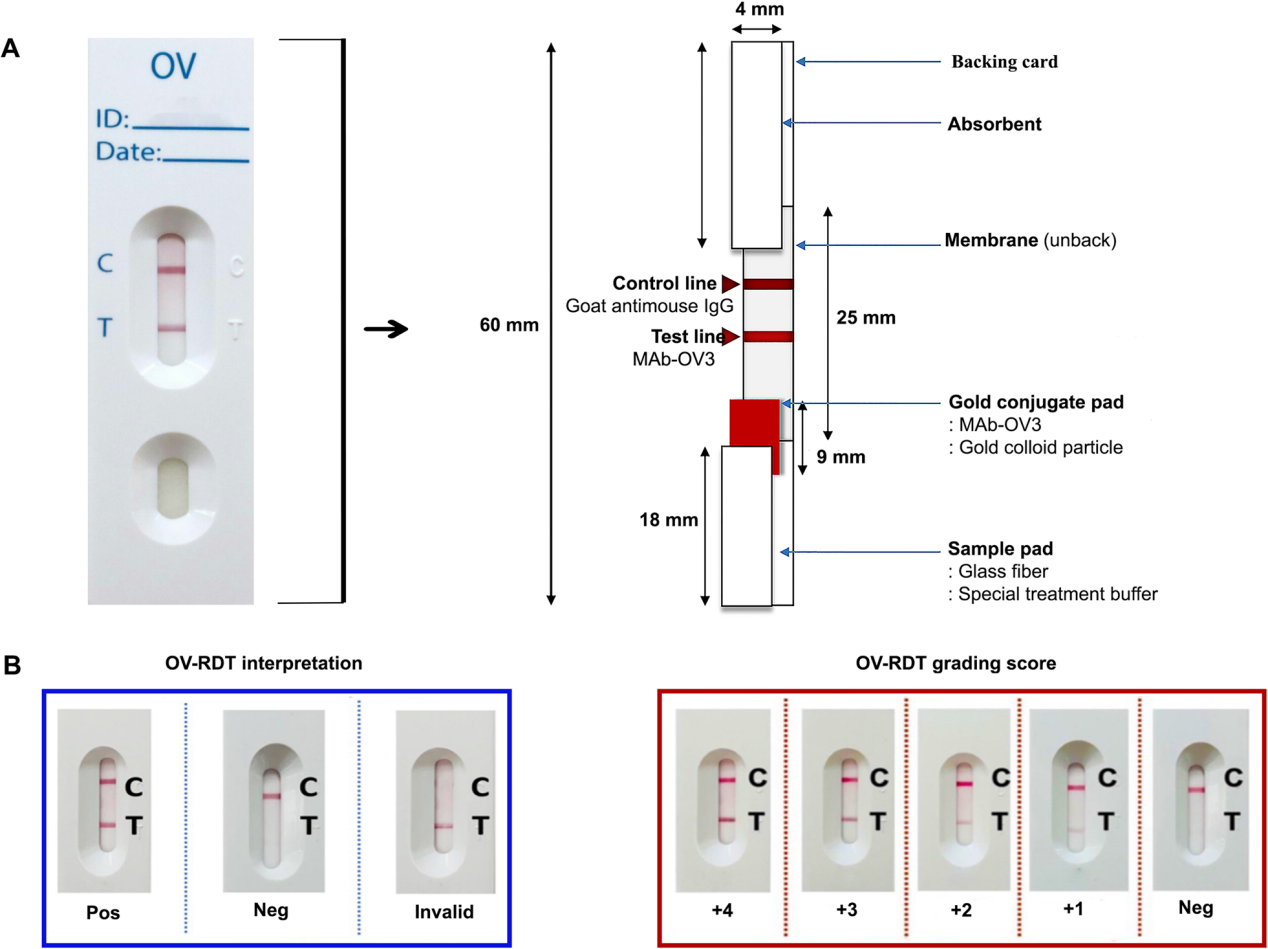
Schematic diagram of urinary OV-RDT for the diagnosis of opisthorchiasis (**A**). OV-RDT interpretation and grading score (**B**). *mAb* monoclonal antibody, OV-RDT *Opisthorchis viverrine*-rapid diagnosis test. The color intensity at T was expressed as + 4 for the highest intensity and + 1 for the lowest intensity

The structure of OV-RDT was based on immunochromatographic lateral flow methodology, which is standard with in vitro diagnostic medical devices (IVDs). The test cassette consisted of a series of membranes, test (T) and control (C) lines. The key component is the specific monoclonal antibody to *O. viverrini* antigen (clone OV-3), and the reaction is signalled by a nanogold particle anti-mouse IgG conjugate. OV-RDTs were produced in collaboration with the National Center for Genetic Engineering and Biotechnology, and K Bioscience Ltd, Thailand. The mAb and anti-mouse IgG (Lampire Biological Laboratories, Pipersville, PA, USA) were dispensed onto nitrocellulose membranes (Sartorius Stedim Biotech SA, Goettingen, Germany) to serve as the T and C lines, respectively. The optimal conditions were as follows: 1 mg/ml of goat anti-mouse IgG was absorbed at the control line, 2 mg/ml of mAb was absorbed at the test line, and 10 μg/ml of mAb conjugated with colloidal gold was sprayed onto a glass microfiber filter GF33 (conjugate pad) (Whatman Schleicher & Schuell, Dassel, Germany) (Fig. 1).

The test was performed by applying three drops of urine sample (120 μl) and one drop of buffer (40 μl) in the receiving bay then allowing the reaction to occur over 10 min to obtain test results. For interpreting the results, if there were two reactive bands of both T and C lines, the test was positive for opisthorchiasis. If there was one band at the control line, the test was negative for opisthorchiasis. The control line should always appear, indicating test validity. The interpretation of the urinary OV-RDT result was based on the intensity of T-band. The grading scores from 2 independent assessors were compared to finalize the results. The inconsistencies of faint/indecisive band were considered negative. The standard OV-RDT colour chart for the grading score based on the band intensity from 1–4 was constructed by using *O. viverrini* crude antigen diluted in clean urine beginning from 5000 ng/ml (+ 4) and diluted to 28.5 ng/ml (+ 1) (Fig. 1).

### Limit of detection (LOD) for urinary antigen ELISA and OV-RDT

Crude somatic antigens were spiked in negative urine specimens by starting from 10 µg/ml to 5000 µg/ml for LOD analysis by ELISA and urinary OV-RDT.

### Statistical analysis

Data were analysed by using SPSS version 22 (International Business Machines, USA). The McNemar chi-square test was used to determine the significance of differences in proportions for prevalence by diagnostic testing. The Kruskal–Wallis tests were used to assess the correlation between antigen concentration and the grading score of urinary OV-RDT. The performance of OV-RDT for sensitivity, specificity, and predictive values was calculated by using faecal FECT as a reference standard. Analysis of diagnostic agreements between tests was measured by Cohen’s kappa coefficient (*κ*-value) and was interpreted using kappa value guidelines: *κ* < 0 indicated no agreement, 0 to 0.2 indicated poor agreement, 0.21 to 0.4 indicated fair agreement, 0.41 to 0.6 indicated moderate agreement, 0.61 to 0.8 indicated good agreement, and 0.81 to 1.0 indicated excellent agreement [29]. Statistical significance was reached when* P* values were > 0.05.

## Results

### Diagnostic performance of urinary OV-RDT with reference to FECT as a reference standard

The performance of urinary OV-RDT showed 94.2% sensitivity and 93.2% specificity (*n* = 493). The positive predictive values and negative predictive values were 93.1% and 94.3%, respectively (Table 2). The positive rate by OV-RDT was 48.9% and that by FECT was 49.3%. Within FECT-positive samples, 14 (5.7%) were negative by urinary OV-RDT. Out of 250 faecal-negative samples, 17 (6.8%) were positive by urinary OV-RDT. The performance of urine-ELISA showed 100% sensitivity and 93.6% specificity. The positive predictive values and negative predictive values were 100.0% and 96.8%, respectively (Table 2). The positive rate by ELISA was 52.5%. Among the positive ELISA results, the detection rate by urinary OV-RDT was 93.8%.

**Table 2:** Diagnosis of opisthorchiasis by urinary OV-RDT and urinary antigen ELISA and the reference standard FECT (*n* = 493)

	FECT positive	FECT negative	Total
OV-RDT positive	229	17	246
OV-RDT negative	14	233	247
Total	243	250	493
ELISA positive	243	16	259
ELISA negative	0	234	234
Total	243	250	493

*OV-RDT* *Opisthorchis viverrini*-rapid diagnostic test; *FECT* Formalin-ethyl acetate concentration technique; *ELISA* Enzyme-linked immunosorbent assay

### Relationship of urinary OV-RDT with intensity of infection (EPG)

**Fig. 2:**
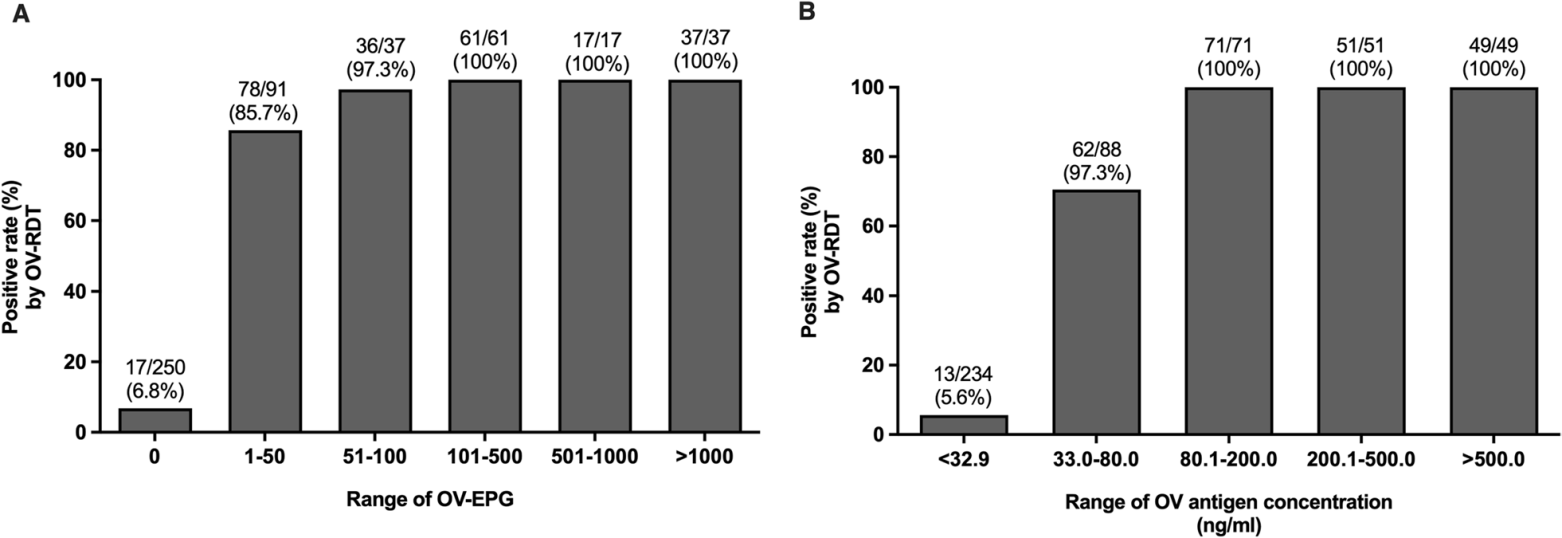
Relationship between diagnosis by urinary OV-RDT with faecal egg count (egg/gram faeces, EPG) determined by the quantitative formalin-ethyl acetate concentration technique (FECT) (*n* = 493) (**A**) and antigen concentration in urine by ELISA (**B**). The data shown are the percent positive in each egg count group and antigen concentration group. There were significant associations between percent positive by OV-RDT with EPG (*χ*^2^ for trend = 261.5, *P* < 0.0001) and antigen concentration (*χ*^2^ for trend = 298.4, *P* < 0.0001)

Within the paired urine and faecal specimens (*n* = 493), in *O. viverrini* egg-negative cases by FECT (*n* = 250), 17 cases (6.8%) were antigen positive by urinary OV-RDT. Within the egg-positive group (*n* = 243), 229 cases (94.2%) were urinary OV-RDT positive. High percentages of antigen positivity by urinary OV-RDT (85.7–100%) were seen across the intensity of infection (1– > 1000 EPG) (Fig. 2). The positive rate by OV-RDT was significantly related to increasing EPG (*χ*^2^ = 381.2, *P* < 0.0001), but no difference was detected between EPG 1–50 and 51–100 (*P* > 0.05) (Fig. 2). Moreover, antigen concentrations in urine were significantly correlated with the positive rate of OV-RDT (*χ*^2^ = 370.6, *P* < 0.0001).

### Agreement between tested methods

The test for agreement between FECT versus urinary OV-RDT and urinary antigen ELISA versus urinary OV-RDT revealed excellent agreement. Kappa values (*κ*) were 0.874 and 0.842, respectively. The kappa tests showed excellent agreement (*κ* = 0.935) between FECT and Urine-ELISA (Table 3).

**Table 3:** Diagnostic agreements between methods in opisthorchiasis, including FECT, urinary OV-RDT and urinary antigen ELISA

OV-RDT	Kappa	95% *CI*		*P*-value
		Lower	Upper	
FECT vs OV-RDT	0.874	0.831	0.917	< 0.001
Urine-ELISA vs OV-RDT	0.842	0.794	0.889	< 0.001
FECT vs Urine-ELISA	0.935	0.903	0.966	< 0.001

The data shown are kappa values, 95% confidence intervals (95% *CI*s) and *P* values

*OV-RDT* *Opisthorchis viverrini*-rapid diagnostic test; *ELISA* Enzyme-linked immunosorbent assay; *FECT* Formalin-ethyl acetate concentration technique

### Limits of detection

The limits of detection of crude somatic *O. viverrini* antigen-spiked urine samples by urinary OV-RDT were 28.5 ng/ml (Additional file 1A). The limit of detection of crude antigen-spiked urine samples by urinary antigen ELISA was 78 ng/ml (Additional file 1B).

### Cross-reactivity test of urinary OV-RDT

A separate set of participants with parasite infections other than *O. viverrini* determined by faecal examination (FECT) were analysed for cross reactivity with urinary OV-RDT. Positive tests by urinary OV-RDT were found in 1 of 8 subjects infected with minute intestinal fluke and one of four subjects infected with *Trichuris trichiura.* No reaction was found in subjects infected with *Strongyloides stercoralis*, *Taenia* spp., *Echinostoma* spp. and hookworms. Overall, the cross reaction was found in two out of six species of helminths, with a rate of 2% from 96 individuals.

### Field trial of urinary OV-RDT and urinary antigen ELISA

Study participants (*n* = 1629), originating from Kalasin (KSN), Mahasarakham (MSK) and Roi Et (RET) in northeast Thailand, were enrolled and screened for *O. viverrini* infection by both urinary OV-RDT and urinary antigen ELISA. Based on OV-RDT, 866 cases (53.2%) were positive for *O. viverrini* antigen. Within the OV-RDT positive group, 815 out of 866 cases (94.1%) were *O. viverrini* antigen positive by urinary antigen ELISA (Additional file 2).

The baseline demographic characteristics and status of *O. viverrini* infection of the sample participants for praziquantel treatment are shown in Table 4. Among 1629 subjects who provided urine samples, 866 (53.2%) and 879 subjects (54.0%) were positive for *O. viverrini* antigen by urinary OV-RDT and urinary antigen ELISA, respectively. The highest prevalence of *O. viverrini* infection by urinary OV-RDT was in participants from RET (67.4%), whereas moderate and low rates were found in participants from MKM (37.8%) and KSN (32.9%), respectively. The overall prevalence of *O. viverrini* infection by urinary OV-RDT was 368 (22.6%) males and 498 (30.6%) females. The age-prevalence profiles of *O. viverrini* by OV-RDT and urine ELISA revealed a similar pattern, where the prevalence ranged from 4–21% and peaked at age 51–60 years.

**Table 4:** Status of *Opisthorchis viverrini* infection at baseline study diagnosed by urinary OV-RDT and urinary antigen ELISA in relation to age and sex of the participants

Factor	OV-RDT				ELISA			
	KSN (*n* = 322) No. +ve (%)	MKM (*n* = 408) No. +ve (%)	RET (*n* = 899) No. +ve (%)	Total (*n* = 1629) No. +ve (%)	KSN (*n* = 322) No. +ve (%)	MKM (*n* = 408) No. +ve (%)	RET (*n* = 899) No. +ve (%)	Total (*n* = 1629) No. +ve (%)
Gender								
Males, *n* (%)	41 (12.7)	50 (12.3)	277 (30.8)	368 (22.6)	41 (12.7)	55 (13.5)	281 (31.3)	377 (23.1)
Females, *n* (%)	65 (20.2)	104 (25.5)	329 (36.6)	498 (30.6)	64 (19.9)	111 (27.2)	327 (36.4)	502 (30.8)
Total	106 (32.9)	154 (37.8)	606 (67.4)	866 (53.2)	105 (32.6)	166 (40.7)	608 (67.6)	879 (54.0)
Age range, *n* (%)								
< 40 years	7 (2.2)	27 (6.6)	38 (4.2)	72 (4.4)	6 (1.9)	26 (6.4)	37 (4.1)	69 (4.2)
40–50 years	36 (11.2)	35 (8.6)	194 (21.6)	265 (16.3)	35 (10.9)	39 (9.6)	192 (21.4)	266 (16.3)
51– 60 years	44 (13.7)	53 (13.0)	247 (27.5)	344 (21.1)	42 (13.0)	55 (13.5)	252 (28.0)	349 (21.4)
> 60 years	19 (5.9)	39 (9.6)	127 (14.1)	185 (11.4)	22 (6.8)	46 (11.3)	127 (14.1)	195 (12.0)
*χ*^2^ test for trend (*P*-value)	0.381	0.689	< 0.001	0.137	0.939	0.643	0.0004	0.011

*OV-RDT* *Opisthorchis viverrini*-rapid diagnostic test; *ELISA* Enzyme-linked immunosorbent assay; *KSN* Kalasin; *MKM* Maha Sarakham; *RET* Roi Et

The prevalence of *O. viverrini* by urinary OV-RDT (*n* = 1629) had a significant positive association with a range of antigen concentrations in urine (*χ*^2^ = 370.6, *P* < 0.001) (Additional file 3). The prevalence was low (6.8%) in the antigen-negative range and high (88.8–99.8%) in the antigen-positive range. Based on the urinary OV-RDT grading score (0, + 1, + 2, + 3, + 4), 7.8% positive rates were seen in grading score 0 and 89.8–99.1% in grading score + 1 to + 4 by ELISA (Additional file 3). There was a positive correlation between antigen concentration in urine and the grading score of urinary OV-RDT (Kruskal‒Wallis test = 316.2, *P* < 0.0001).

**Fig. 3:**
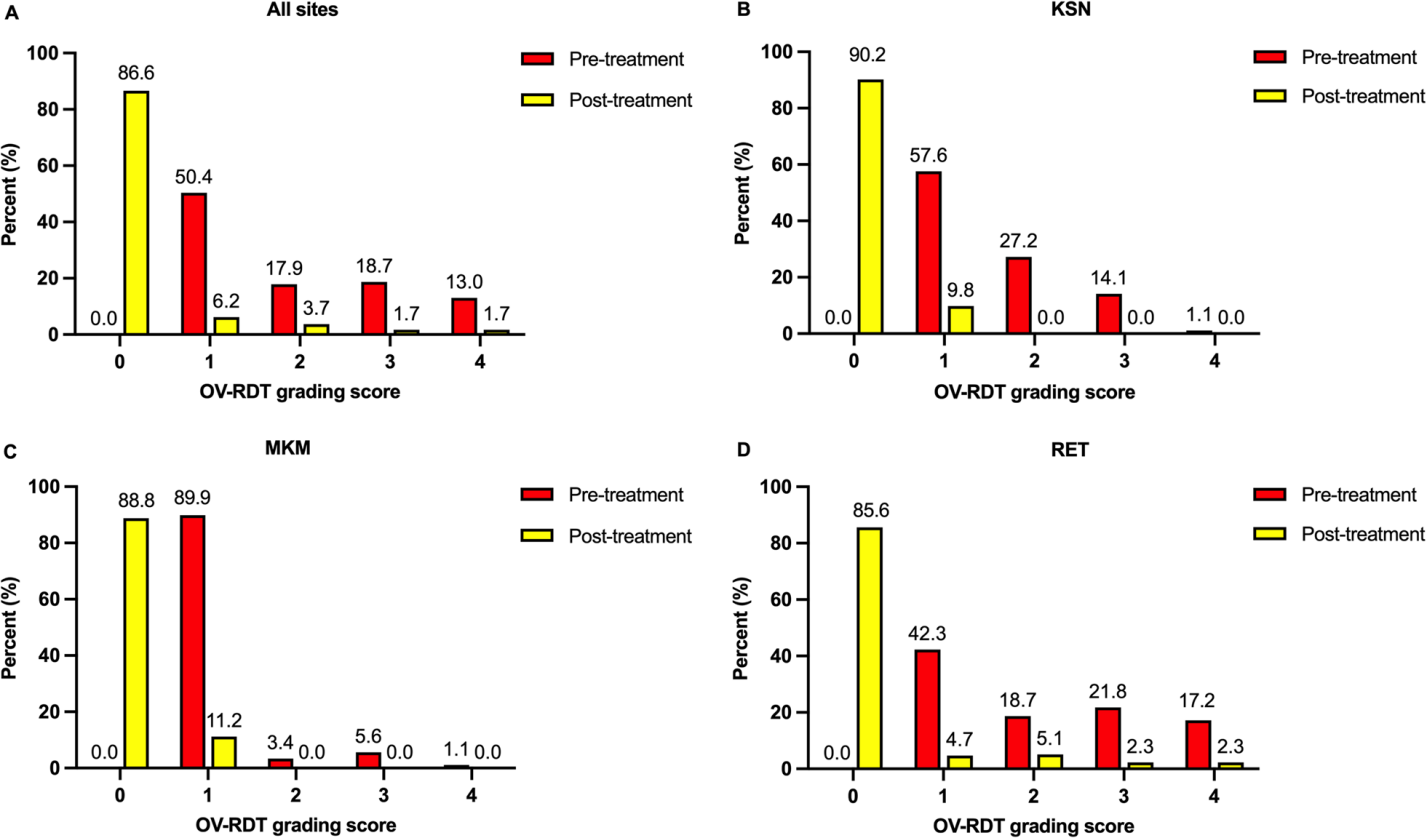
Distributions of urinary OV-RDT grading scores at pre- and post-treatment in all sites (**A**), KSN (**B**), MKM (**C**), and RET (**D**). The bars represent the percent of participant in each grading score of urinary OV-RDT at pre- (red) and post-treatment (yellow). KSN Kalasin, MKM Maha Sarakham, RET Roi Et

**Fig. 4:**
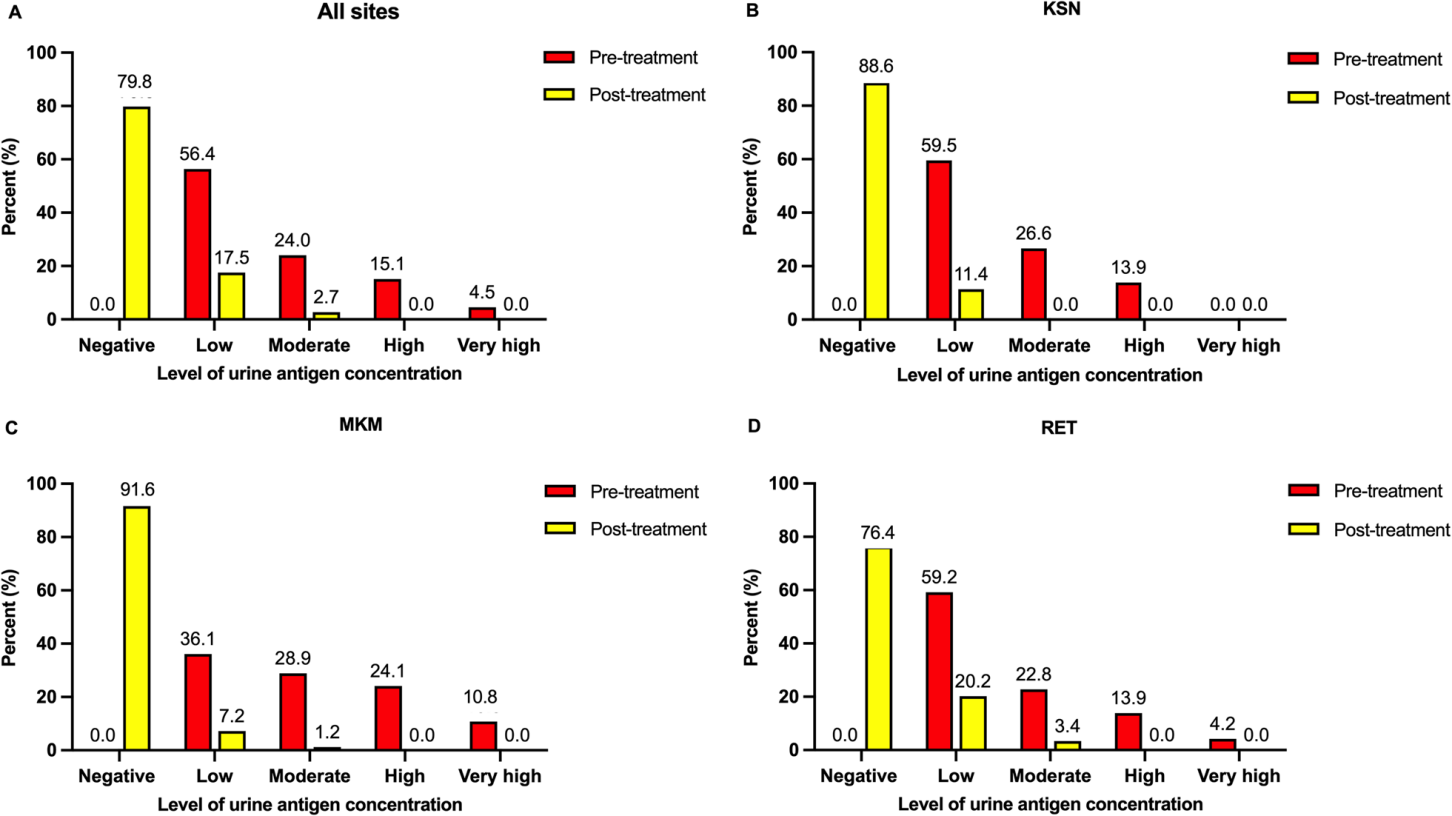
Distributions of *Opisthorchis viverrini* antigen concentration in urine by urinary antigen ELISA at pre- and post-treatment with praziquantel in combined all 3 sites (**A**), KSN (**B**), MKM (**C**) and RET (**D**). Negative antigen groups were individuals with antigen concentration below 32.9 ng/ml in urine, light intensity groups were individuals with antigen concentration between 32.9–80.0 ng/ml in urine, moderate intensity groups were individuals with antigen concentration between 80.1–200.0 ng/ml, high intensity groups were individuals with antigen concentration > 80 ng/ml in urine. The bars represent the percent of participant in each concentration group at pre-(red) and post-treatment (yellow). KSN Kalasin, MKM Maha Sarakham, RET Roi Et

At 1 month after praziquantel anthelminthic treatment, the cure rates were 85.6–90.2% and 76.8–90.2%, determined by urinary OV-RDT and urinary antigen ELISA, respectively. The overall cure rates estimated by urinary OV-RDT (86.6%) and by urinary antigen ELISA (80.5%) were not different (*χ*^2^-test*, P* > 0.05) (Additional file 4). There were significant reductions in antigen concentration following treatment in both the cured and non-cured group (Wilcoxon signed rank test = − 141,178 vs. − 4078*, P* < 0.001, Additional file 5). There were similar patterns in frequency distributions of test results at baseline and after treatment in which the test results moved toward negative and low grading scores for urinary OV-RDT and urinary antigen ELISA after treatment (Figs. 3 and 4).

## Discussion

The recent availability of urinary antigen assays has allowed significant progress in the diagnosis of opisthorchiasis away from traditional faecal examination with its technical complications [12, 21]. This now allows convenient sample collection and handling for opisthorchiasis assessment [22]. To further exploit the utility of urinary assays, this study described the development of OV-RDT to simplify urinary testing and provide point-of-care-test (POCT) for large-scale screening of opisthorchiasis.

The diagnostic performance of urinary OV-RDT had lower sensitivity but similar specificity compared to urinary antigen ELISA. When compared to faecal FECT, the positivity rates of urine antigen detection, both by urinary OV-RDT and urinary antigen ELISA, correlated positively with the intensity of infection and showed high diagnostic agreements. The high diagnostic concordance and the correlation of urinary OV-RDT grading scores and intensity of infection in terms of faecal egg count and antigen concentration in urine lend support to the reliability of urinary OV-RDT.

Antigen detection in urine by urinary antigen ELISA is a more sensitive assay than urinary OV-RDT because the ELISA procedure has several laboratory steps, but it requires longer times, specific laboratory equipment, and also trained laboratory technicians [21]. The OV-RDT is a simplified test platform which has several advantages over an ELISA for antigen detection in urine in opisthorchiasis. Hence, the urinary OV-RDT serves as a practical tool in opisthorchiasis eradication programs requiring onsite assessment far from traditional laboratories.

In the field trial study, the age and sex prevalence profiles obtained from urinary OV-RDT were similar to those obtained by urinary antigen ELISA. Similar to the diagnostic study, there was qualitative and quantitative concordance between diagnosis by urinary OV-RDT and ELISA. In the follow-up study, the overall cure rates from praziquantel treatment in those previously antigen-positive by urinary OV-RDT were not different when subsequently assessed by either urinary OV-RDT or ELISA [30].

In terms of the specificity of urinary OV-RDT, cross-reactivity with urine from *O. viverrini*-negative individuals with other endemic parasitic infections was encountered in a small number of subjects (2%) infected with MIF and *Trichuris trichiura*. The results were fewer than the previous report by urine ELISA for other helminthic parasites, such as *S. stercoralis*, echinostomes, hookworms and *Taenia* spp. [21]. The finding of cross reactivity of urinary OV-RDT to other parasites, particularly MIF is problematic and required consideration. In northeast Thailand, the MIF were mostly lecithodendriid trematodes, i.e., *Phaneropsolus* and *Prosthodendium* which can be separated by egg morphology from *O. viverrini* [31, 32]. In contrast, the eggs from another group of small trematodes such as *Haplorchis* spp. are indistinguishable from *O. viverrini*, and thus require adult worms for identification [33]. Nevertheless, the observed cross reactions of urinary OV-RDT in our study may due to the presence of *O. viverrini* worm but no eggs in faeces were detected [14, 26]. Obviously, DNA detection by PCR will assist for confirmation as previously reported [34]. Moreover, further tests of urine samples infected with parasites from nonendemic areas of opisthorchiasis are needed.

It is likely that antigens produced by worms in the bile ducts pass across the biliary epithelium into the circulation and are filtered through the kidneys before being excreted in urine. To support this, *O. viverrini* antigens have been detected in inflamed biliary tissue after drug treatment in hamster models [35]. Of note, the presence of *O. viverrini* antigen was demonstrated in *O*. *viverrini*-infected hamsters with diseased kidneys [36, 37] and in patients with chronic kidney disease (CKD) [38]. However, the sensitivity and specificity of urinary OV-RDT needs to be further assessed in light of CKD.

Although offering promising new potential, since antigen concentration in faeces correlated with those in urine, additional copro-antigen detection methods should be undertaken to validate urinary OV-RDT performance. Second, it is not known whether *O*. *viverrini* antigens detected in urine are in the form of immune complexes or free antigens. Additional studies are required to elucidate this. Furthermore, the 4-week follow-up period after praziquantel treatment may not be an optimal period for the assessment of urinary OV-RDT performance, and a longer duration may be needed.

## Conclusions

The new urinary OV-RDT developed in this study demonstrated high diagnostic performance for opisthorchiasis. The urinary OV-RDT had almost perfect diagnostic agreement with urinary antigen ELISA and showed significant quantitative correlations with faecal egg count and *O. viverrini* antigen concentrations in urine. In the field application of urinary OV-RDT, it showed similar prevalence of opisthorchiasis and cure rates following praziquantel treatment compared to urinary antigen ELISA. The ease of sample collection, handling and public acceptance compared to faecal collection makes urinary OV-RDT a promising potential POCT method to facilitate large-scale screening and control for the elimination of opisthorchiasis, leading to a reduction in cholangiocarcinoma in Southeast Asia and The Lower Mekong Basin countries.

### Supplementary Information

**Additional file 1. **Limit of detection of OV crude antigen spiked urine samples by urinary OV-RDT (A) and urinary antigen ELISA (B).

**Additional file 2. **Flowchart of study participants in group 3 for follow up study.

**Additional file 3. **Relationship between diagnostic results of urinary OV-RDT and antigen concentration in urine determined by urinary antigen ELISA in opisthorchiasis. Positive rates of urinary OV-RDT and antigen concentration (A) and positive rates by urinary antigen ELISA and grading score of urinary OV RDT (B).

**Additional file 4. **Cure rate of *Opisthorchis viverrini* infection after praziquantel treatment determined by urinary OV-RDT and urinary antigen ELISA.

**Additional file 5. **Comparisons of antigen concentrations participants infected with *Opisthorchis viverrini* determined by urinary antigen ELISA between pre- and post-treatment with praziquantel (40 mg/kg body weight). The right panel showed cured (A) and uncured participants (B) in combined study site.

## Data Availability

The datasets used and/or analysed during the current study are available from the corresponding author on reasonable request.

## Abbreviations

OV: *Opisthorchis viverrini*
CCA: Cholangiocarcinoma
FECT: Formalin-ethyl acetate concentration technique
EPG: Eggs per gram faeces
RDT: Rapid diagnostic test
OV-RDT: *Opisthorchis viverrini*-rapid diagnostic test
POCT: Point-of-care-test
ICT: Immunochromatographic techniques
IVDs: In vitro Diagnostic medical devices
T-line: Test line
C-line: Control line
ELISA: Enzyme-linked immunosorbent assay
OD: Optical density
ROC: Receiver operation curve
HAT: Hypoxanthine-aminopterin-thymidine medium
*k*: Cohen’s kappa coefficient
KSN: Kalasin
RET: Roi-et
MKM: Maha Sarakham
