# Supplementary figures and images for: Accuracy of a new rapid diagnostic test for urinary antigen detection and assessment of drug treatment in opisthorchiasis
Source: Infect Dis Poverty. 2023 Nov 21;12:102. doi: 10.1186/s40249-023-01162-4 (PMC10662682; doi:10.1186/s40249-023-01162-4)

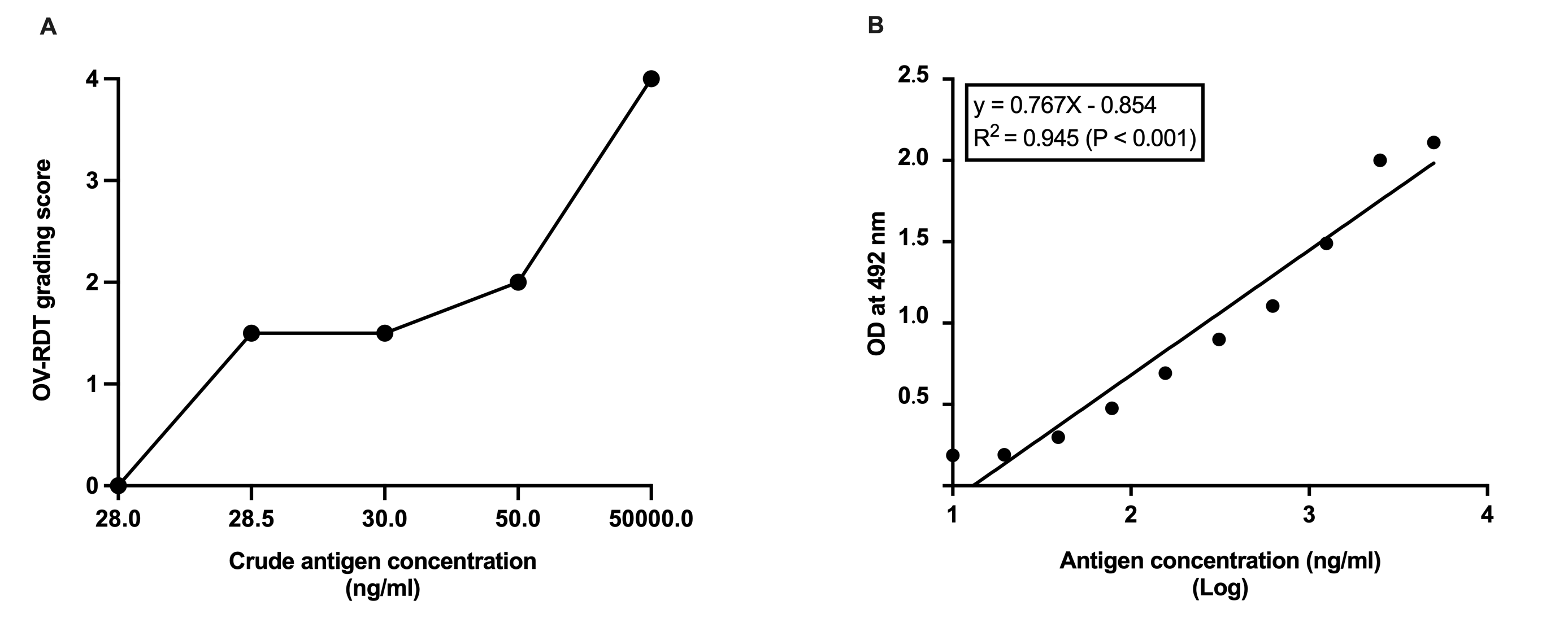

Supplement: Supplementary file 1 — Additional file 1. Limit of detection of OV crude antigen spiked urine samples by urinary OV-RDT (A) and urinary antigen ELISA (B). [file 40249_2023_1162_MOESM1_ESM.tiff]

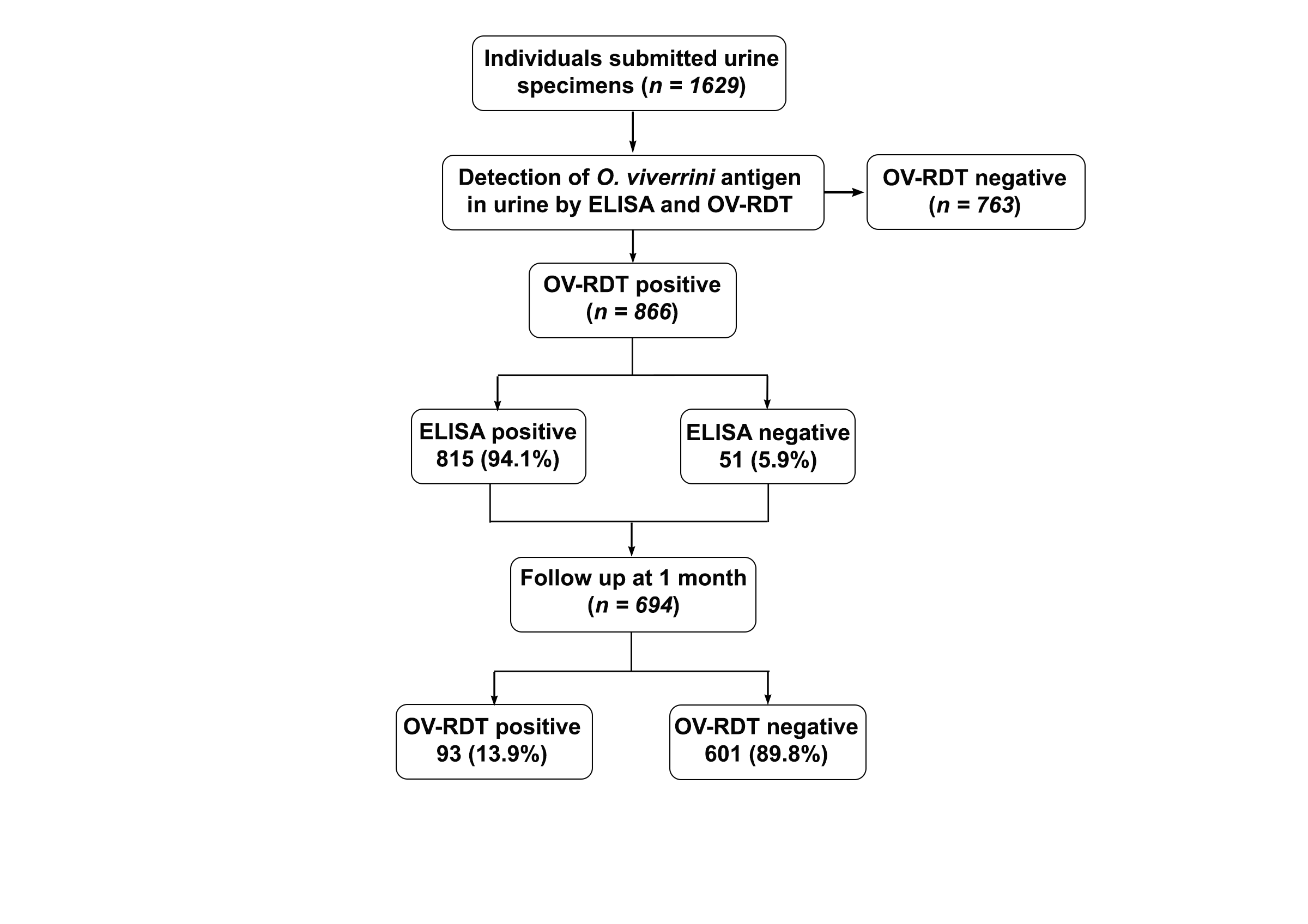

Supplement: Supplementary file 2 — Additional file 2. Flowchart of study participants in group 3 for follow up study. [file 40249_2023_1162_MOESM2_ESM.tif]

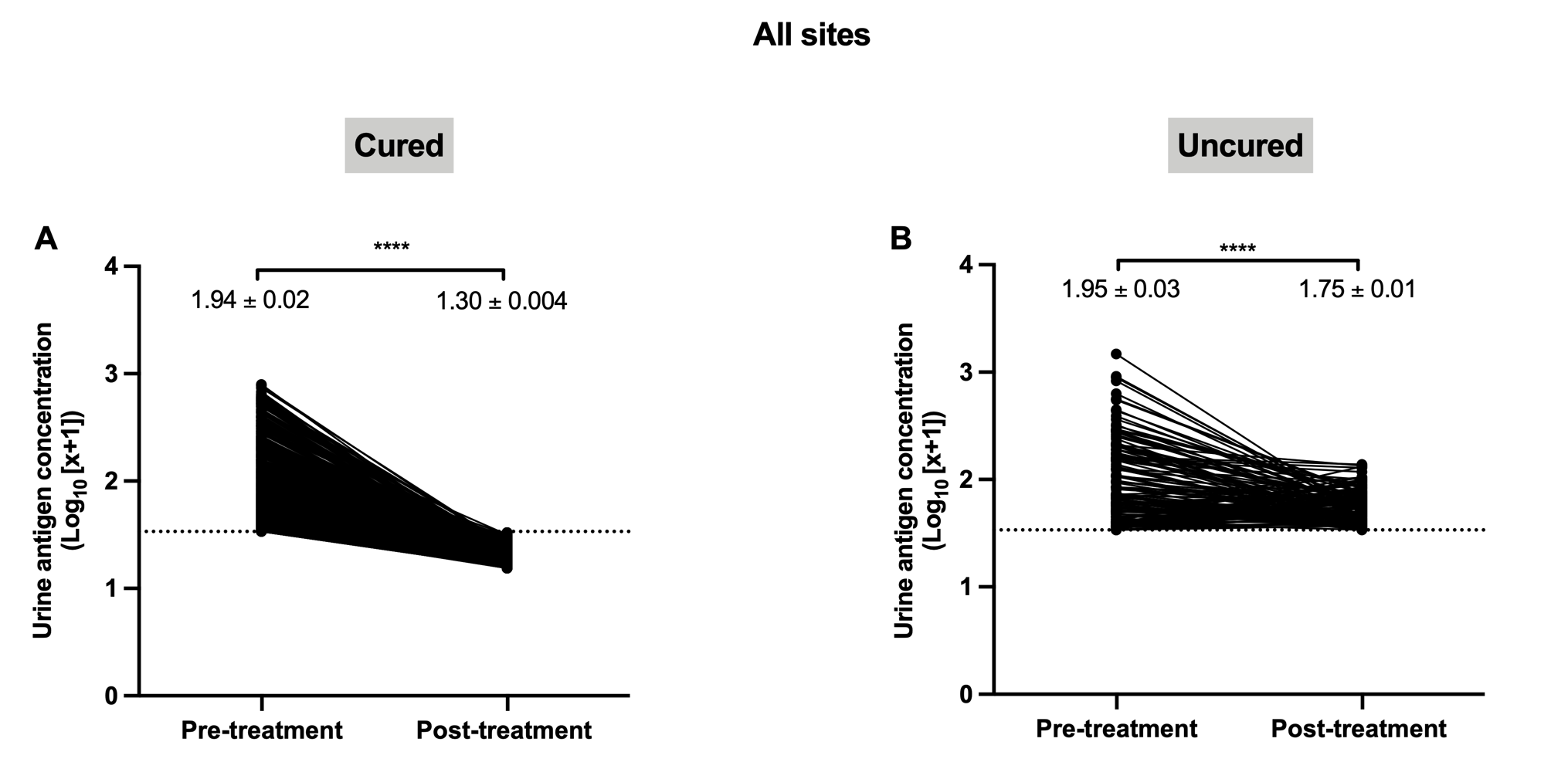

Supplement: Supplementary file 5 — Additional file 5. Comparisons of antigen concentrations participants infected with Opisthorchis viverrini determined by urinary antigen ELISA between pre- and post-treatment with praziquantel (40 mg/kg body weight). The right panel showed cured (A) and uncured participants (B) in combined study site. [file 40249_2023_1162_MOESM5_ESM.tiff]
