# Supplementary material for: Accuracy of a new rapid diagnostic test for urinary antigen detection and assessment of drug treatment in opisthorchiasis
Source: Infect Dis Poverty. 2023 Nov 21;12:102. doi: 10.1186/s40249-023-01162-4 (PMC10662682; doi:10.1186/s40249-023-01162-4)
Supplement: Supplementary file 3 — Additional file 3. Relationship between diagnostic results of urinary OV-RDT and antigen concentration in urine determined by urinary antigen ELISA in opisthorchiasis. Positive rates of urinary OV-RDT and antigen concentration (A) and positive rates by urinary antigen ELISA and grading score of urinary OV RDT (B). [file 40249_2023_1162_MOESM3_ESM.docx]

**Additional file 3** Relationship between diagnostic results of urinary OV-RDT and antigen concentration in urine determined by urinary antigen ELISA in opisthorchiasis. Positive rates of urinary OV-RDT and antigen concentration (A) and positive rates by urinary antigen ELISA and grading score of urinary OV RDT (B).

(A)

| **ELISA** | **Antigen (ng/ml)** | ***N*** | **Positive OV-RDT** | **Positive rate of OV-RDT (95% *CI*)** |
| --- | --- | --- | --- | --- |
| Negative | < 32.9 | 750 | 51 | 6.8 (5.21–8.93) |
| Low | < 80.0 | 509 | 452 | 88.8 (85.76–91.25) |
| Moderate | < 200.0 | 202 | 201 | 99.8 (97.25–99.97) |
| High | > 200.0 | 168 | 162 | 96.4 (92.42–98.35) |
| *P*-value^1^ |  |  |  | < 0.001 |

^1^ Chi-square test

(B)

| **OV-RDT score** | ***N*** | **Positive (*n*) by ELISA** | **Positive rate of ELISA (95% *CI*)** | **Antigen concentration (ng/ml) Mean ± SD (log)** |
| --- | --- | --- | --- | --- |
| 0 | 751 | 59 | 7.8 (6.13–10.00) | 1.32 **±** 0.15 |
| +1 | 460 | 413 | 89.8 (86.67–92.22) | 1.89 **±** 0.41 |
| +2 | 147 | 142 | 96.6 (92.28–98.53) | 1.95 **±** 0.37 |
| +3 | 163 | 158 | 96.9 (93.02–98.68) | 1.94 **±** 0.33 |
| +4 | 108 | 107 | 99.1 (94.94–99.95) | 1.93 **±** 0.28 |
| *P*-value^2^ |  |  |  | < 0.001 |

^2^ Kruskal-Wallis test
