# Supplementary material for: Accuracy of a new rapid diagnostic test for urinary antigen detection and assessment of drug treatment in opisthorchiasis
Source: Infect Dis Poverty. 2023 Nov 21;12:102. doi: 10.1186/s40249-023-01162-4 (PMC10662682; doi:10.1186/s40249-023-01162-4)
Supplement: Supplementary file 4 — Additional file 4. Cure rate of Opisthorchis viverrini infection after praziquantel treatment determined by urinary OV-RDT and urinary antigen ELISA. [file 40249_2023_1162_MOESM4_ESM.docx]

**Additional file 4** Cure rate of *Opisthorchis viverrini* infection after praziquantel treatment determined by urinary OV-RDT and urinary antigen ELISA.

| **Locality** | **No. positive at baseline by OV-RDT** | **OV-RDT** | | **ELISA** | | ***P*-value^1^** |
| --- | --- | --- | --- | --- | --- | --- |
|  |  | **Cured, *n*** | **Cure rate (%)** | **Cured, *n*** | **Cure rate (%)** |  |
| KSN | 92 | 83 | 90.2 | 83 | 90.2 | > 0.05 |
| MKM | 89 | 79 | 88.8 | 82 | 92.1 | < 0.05 |
| RET | 513 | 439 | 85.6 | 394 | 76.8 | < 0.05 |
| **Total** | **694** | **601** | **86.6** | **559** | **80.5** | **> 0.05** |

^1^McNemar’s chi-square test
